# Supplementary material for: Leishmania-infected macrophages release extracellular vesicles that can promote lesion development
Source: Life Sci Alliance. 2020 Oct 29;3(12):e202000742. doi: 10.26508/lsa.202000742 (PMC7652379; doi:10.26508/lsa.202000742)
Supplement: Supplementary file 2 [file LSA-2020-00742_SdataF2.pptx]

## Slide 1
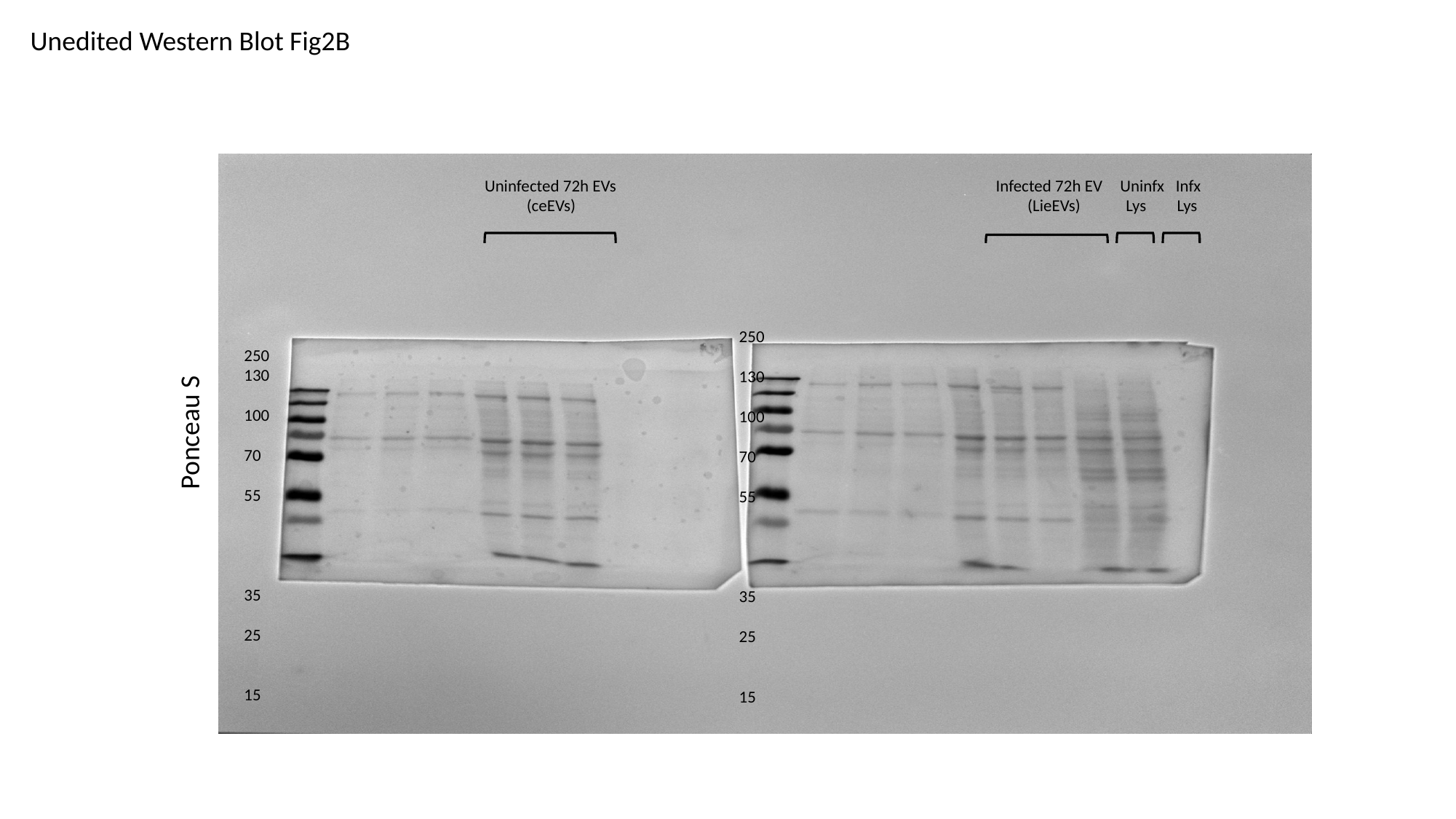

Unedited Western Blot Fig2B
Uninfected 72h EVs Infected 72h EV Uninfx Infx
 (ceEVs) (LieEVs) Lys Lys
250
130
100
70
55
35
25
15
250
130
100
70
55
35
25
15
Ponceau S

## Slide 2
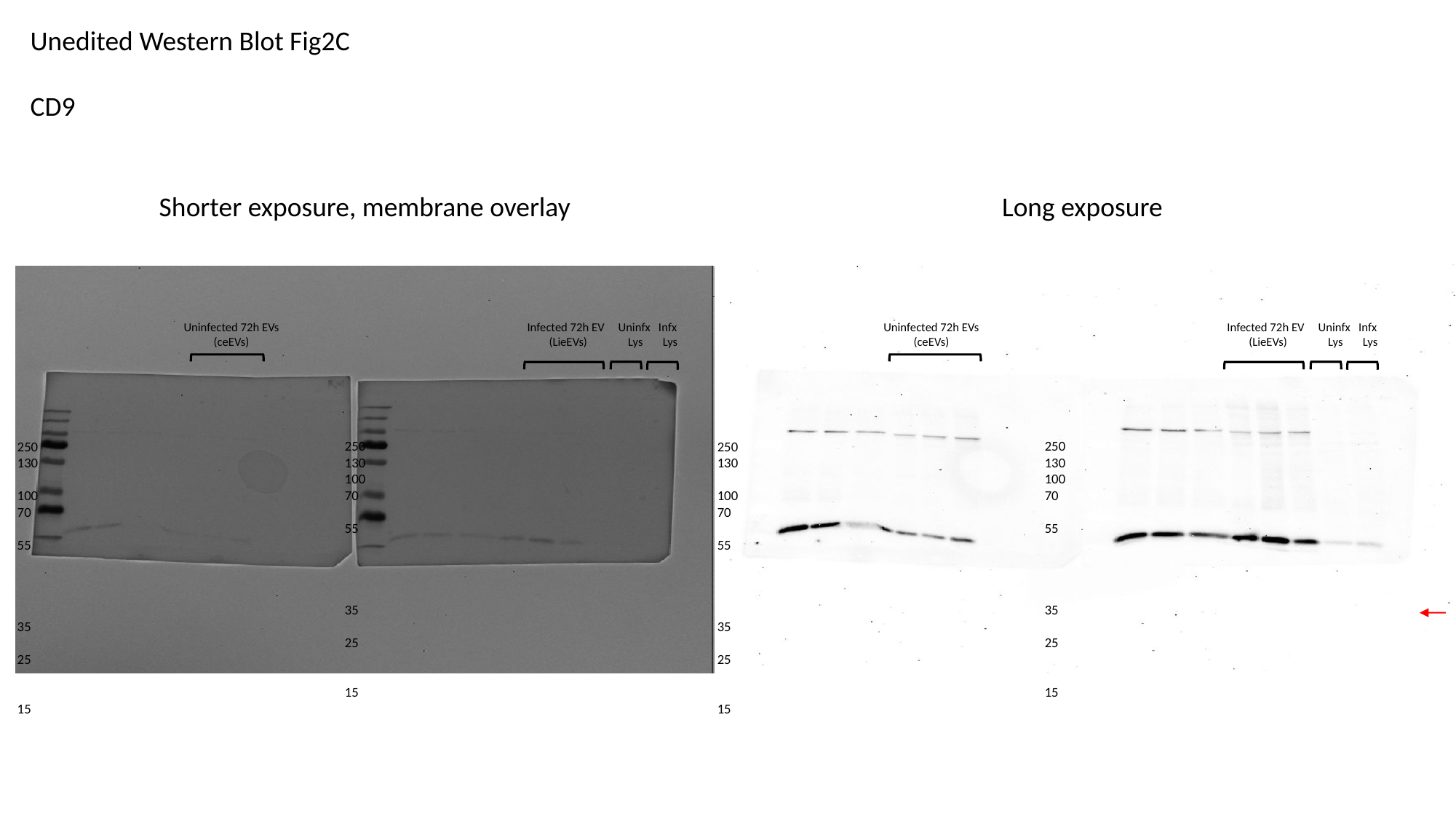

Unedited Western Blot Fig2C
CD9
Shorter exposure, membrane overlay
Long exposure
Uninfected 72h EVs Infected 72h EV Uninfx Infx
 (ceEVs) (LieEVs) Lys Lys
Uninfected 72h EVs Infected 72h EV Uninfx Infx
 (ceEVs) (LieEVs) Lys Lys
250
130
100
70
55
35
25
15
250
130
100
70
55
35
25
15
250
130
100
70
55
35
25
15
250
130
100
70
55
35
25
15

## Slide 3
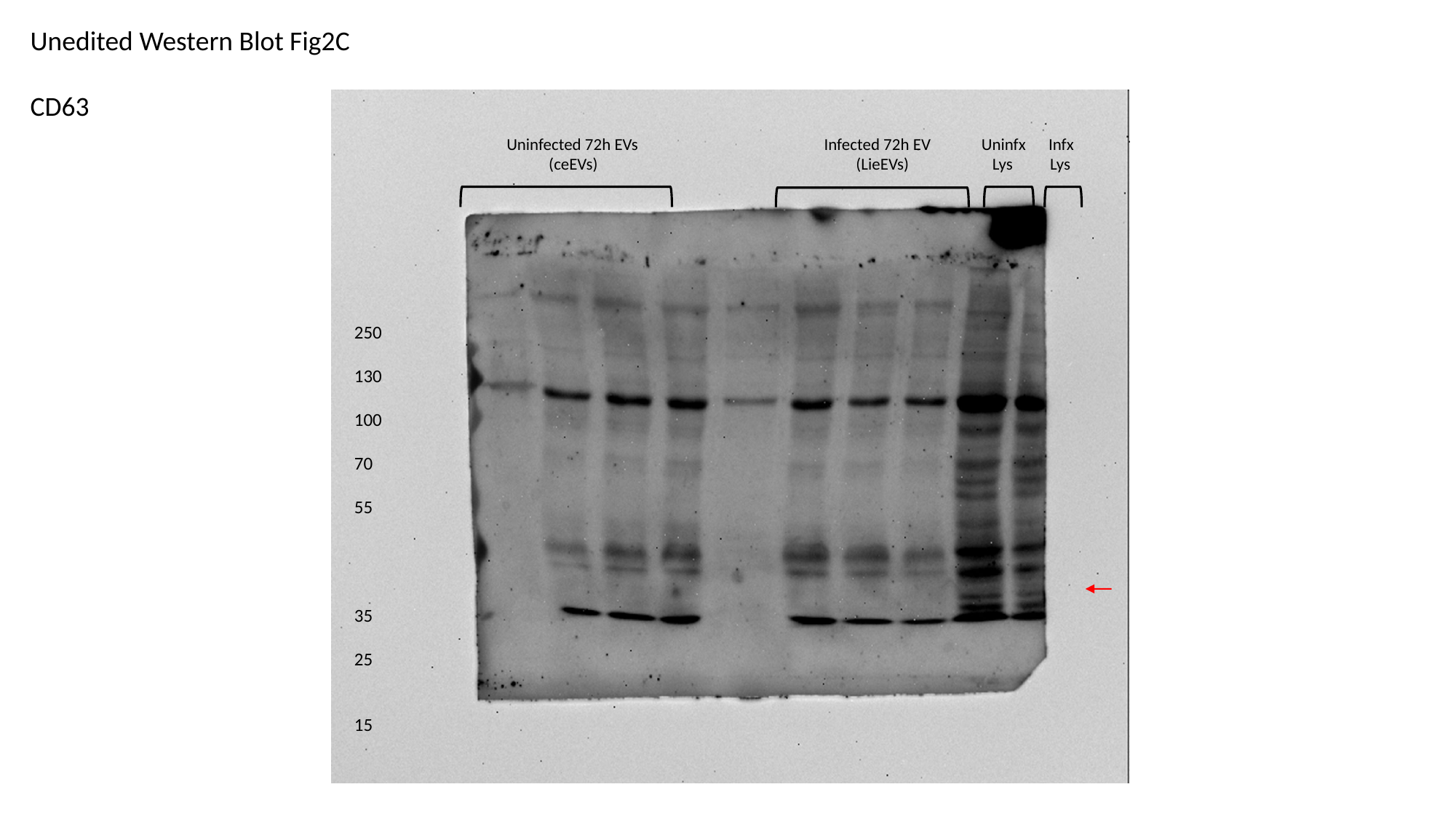

Unedited Western Blot Fig2C
CD63
 Uninfected 72h EVs Infected 72h EV Uninfx Infx
 (ceEVs) (LieEVs) Lys Lys
250
130
100
70
55
35
25
15

## Slide 4
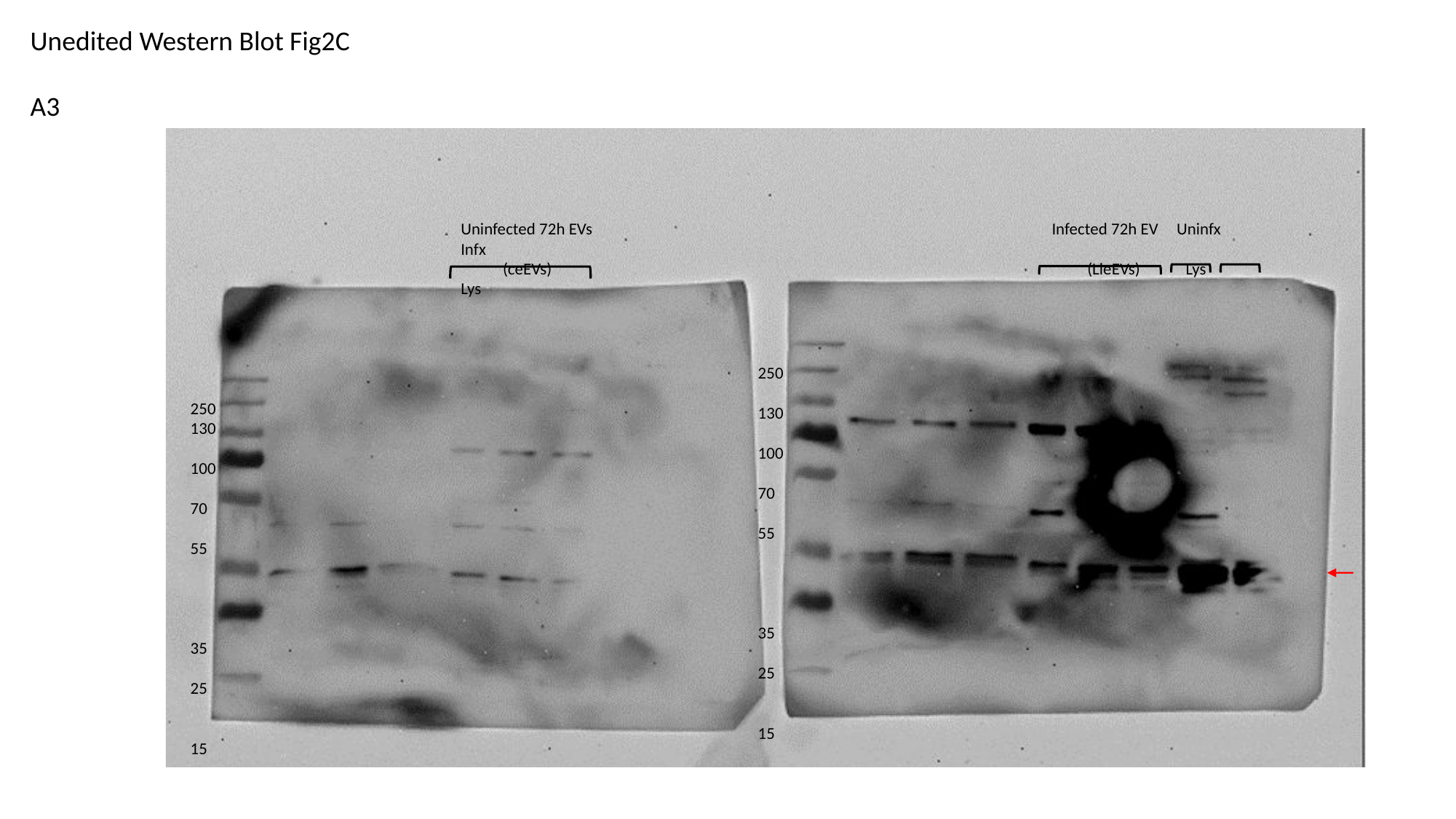

Unedited Western Blot Fig2C
A3
Uninfected 72h EVs Infected 72h EV Uninfx Infx
 (ceEVs) (LieEVs) Lys Lys
250
130
100
70
55
35
25
15
250
130
100
70
55
35
25
15

## Slide 5
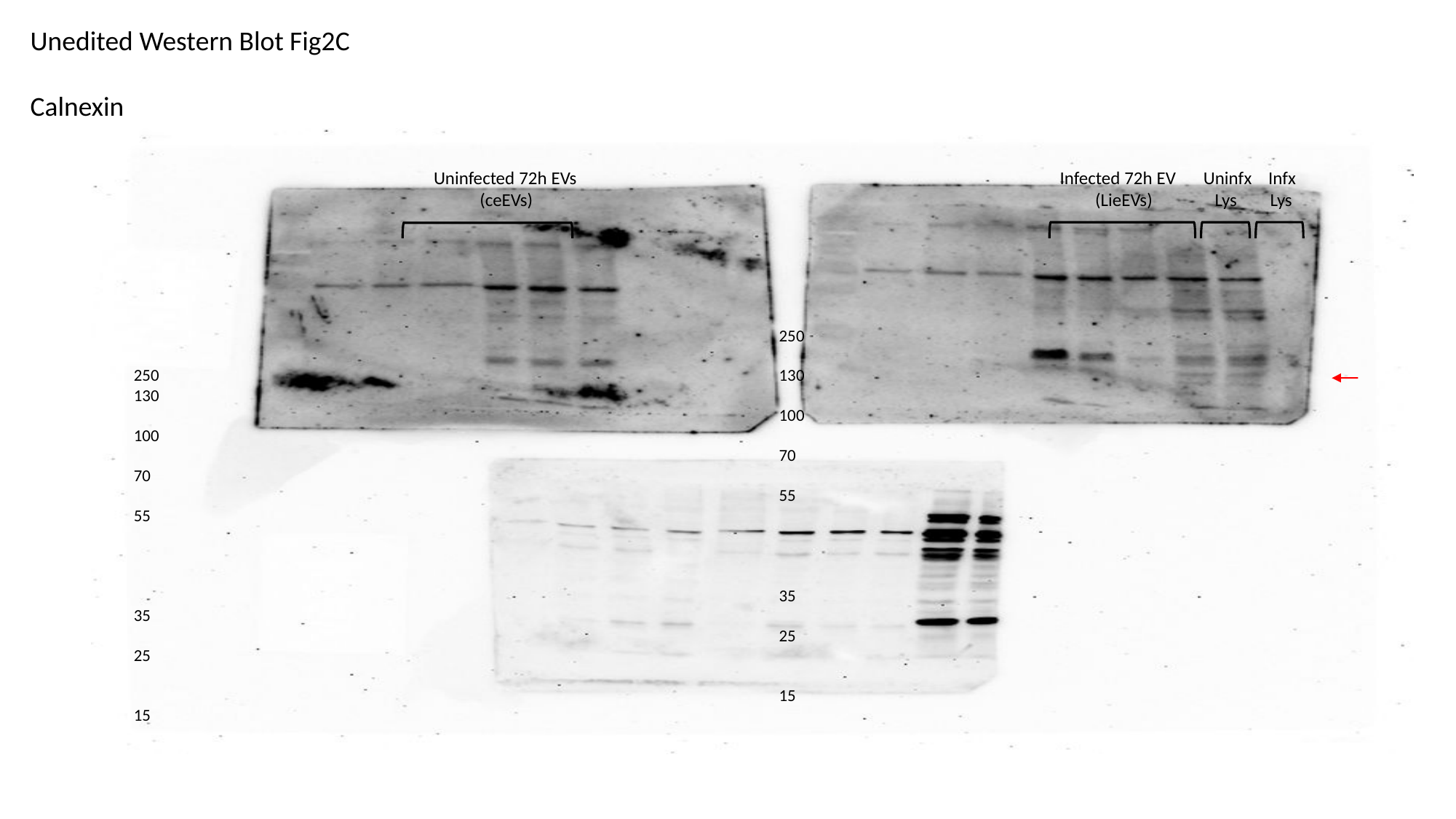

Unedited Western Blot Fig2C
Calnexin
Uninfected 72h EVs Infected 72h EV Uninfx Infx
 (ceEVs) (LieEVs) Lys Lys
250
130
100
70
55
35
25
15
250
130
100
70
55
35
25
15
