# Supplementary material for: Leishmania-infected macrophages release extracellular vesicles that can promote lesion development
Source: Life Sci Alliance. 2020 Oct 29;3(12):e202000742. doi: 10.26508/lsa.202000742 (PMC7652379; doi:10.26508/lsa.202000742)
Supplement: Supplementary file 4 [file LSA-2020-00742_SdataFS5.pptx]

## Slide 1
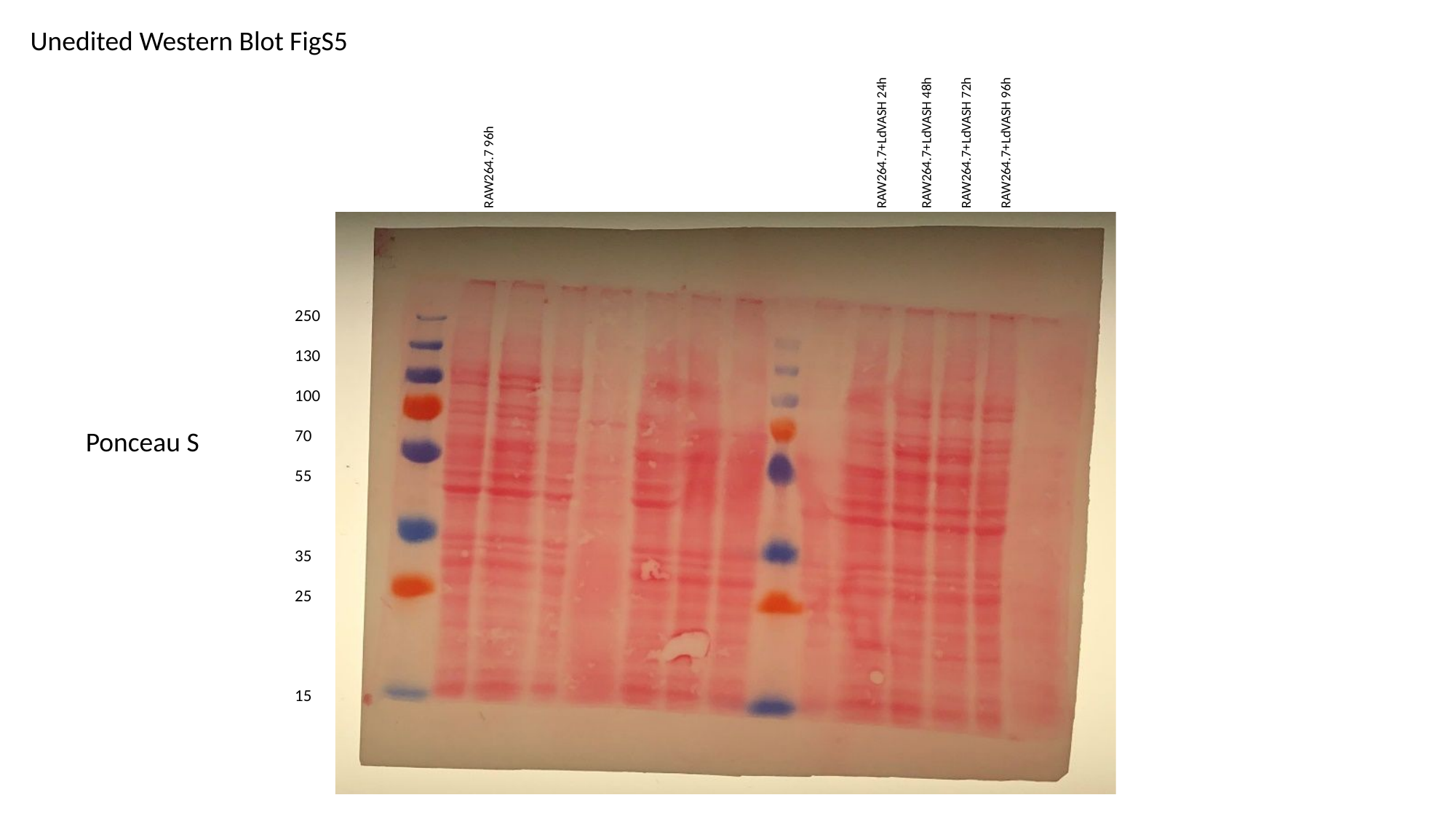

Unedited Western Blot FigS5
RAW264.7 96h
RAW264.7+LdVASH 24h
RAW264.7+LdVASH 72h
RAW264.7+LdVASH 96h
RAW264.7+LdVASH 48h
250
130
100
70
55
35
25
15
Ponceau S
